# Pemphigus vegetans successfully treated with systemic corticosteroid and rituximab: a rare case report with 15-month follow-up and literature review

**DOI:** 10.3389/fimmu.2025.1701439

**Published:** 2026-01-05

**Authors:** Hongyan Ji, Liuqing Chen, Jinbo Chen

**Affiliations:** 1 Department of Dermatology, Wuhan No.1 Hospital, Wuhan, China; 2 Department of Dermatology, Traditional Chinese and Western Medicine Hospital of Wuhan, Tongji Medical College, Huazhong University of Science and Technology, Wuhan, China; 3 Hubei Province & Key Laboratory of Skin Infection And Immunity, Wuhan, China

**Keywords:** pemphigus vegetans, plaques, prednisone, rituximab, steroid myopathy

## Abstract

Pemphigus vegetans (PVeg) is a rare variant of pemphigus vulgaris characterized by pustules and/or vegetating plaques, preferentially affecting the flexural areas. The diagnosis of PVeg depends on clinical grounds, histopathology, and direct immunofluorescence. Systemic corticosteroid is the first-line therapy for PVeg, and immunosuppressive treatments such as azathioprine, mycophenolate mofetil, and intravenous immunoglobulins can be used to improve remission rates. Also, there have been a few reports on the use of rituximab for treating PVeg. Here, we present a rare case of PVeg successfully treated by rituximab and provide the first brief review of the application of rituximab in PVeg. Additionally, this patient experienced muscle weakness after treatment but gradually improved with tapering prednisone, which increased our understanding of steroid myopathy.

## Introduction

Pemphigus vulgaris (PV) is a rare and life-threatening mucocutaneous autoimmune blistering disease. Pemphigus vegetans (PVeg) is a rare clinical variant of PV, making up approximately 1%–2% of cases (1). Its clinical appearance may be polymorphic forms characterized by vesicles, bullae, pustules, and erosions that consequently form vegetating masses. The most characteristic feature is the presence of vegetative plaques, most commonly on flexural surfaces such as the axillae and groin (2). The diagnosis of PVeg is based on clinical grounds, histopathology, and direct immunofluorescence. Histopathologically, PVeg is characterized by epidermal hyperplasia, papillomatosis, acanthosis, and intraepidermal abscesses (1). Treatment of PVeg is similar to PV, and systemic corticosteroid is the first-line therapy. Immunosuppressive treatments such as azathioprine, cyclosporine, cyclophosphamide, and mycophenolate mofetil have been used to improve remission rates. Moreover, many reports have shown that rituximab is a successful treatment for PV (3, 4), while the use of rituximab in PVeg is less frequently reported. Herein, we present a case of PVeg successfully treated by rituximab therapy, which allows a rapid tapering of prednisone doses and ameliorates muscle weakness. In addition, we review the published literature on the application of rituximab in PVeg.

## Case presentation

**Figure 1:**
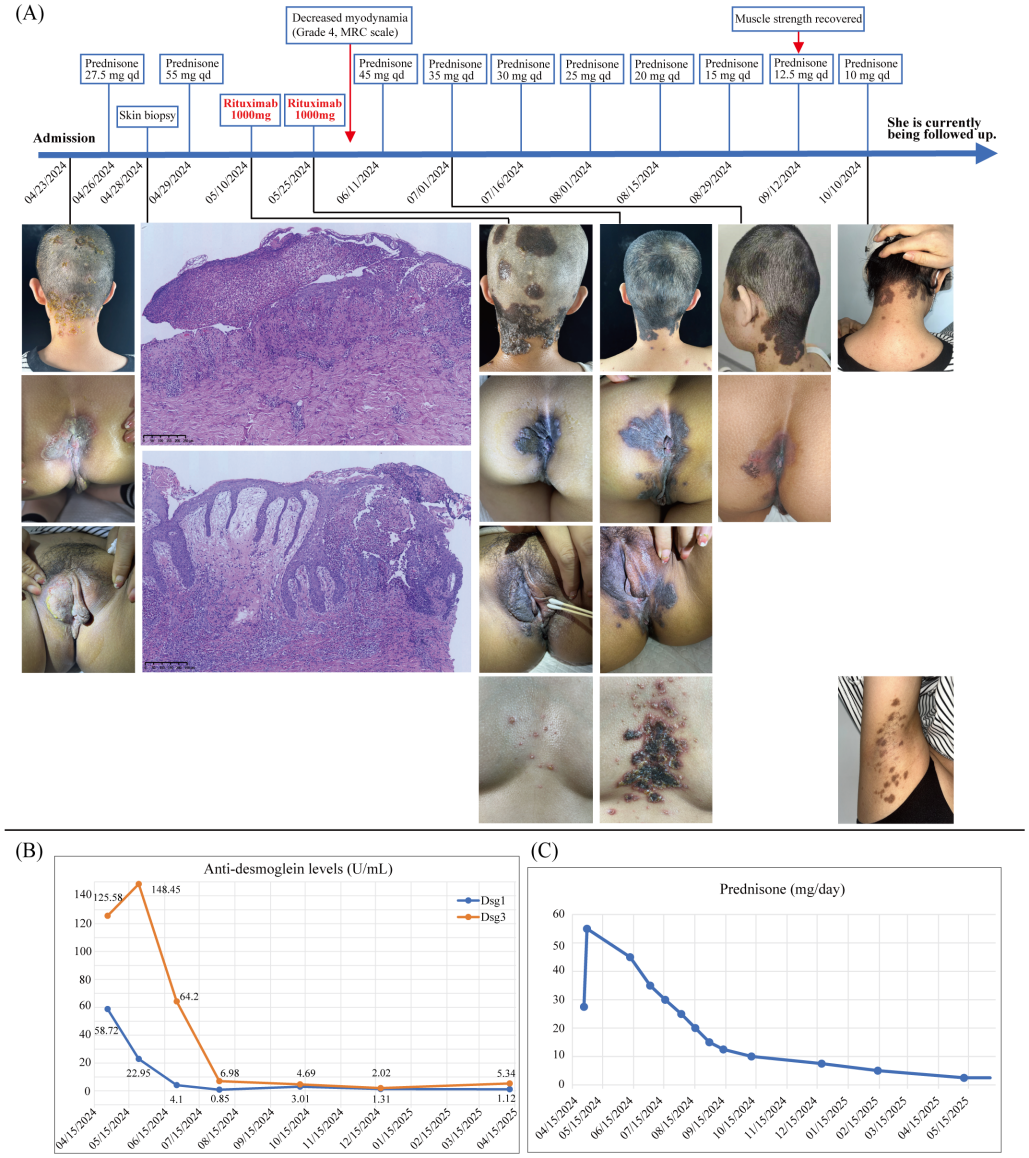
**(A)** Patient timeline demonstrates the therapeutic schedule and disease status. The upper part shows the treatment strategy at different time points. The lower part shows clinical manifestations of the patient over time and skin histopathology of the scalp and perianal region (10×), which shows epidermal hyperplasia, acanthosis, formation of intraepidermal eosinophilic microabscesses, intraepidermal acantholysis with acantholytic cells, and infiltration of inflammation cells around the superficial vessels in the dermis. Papulovesicles, pustules, and erosions on her scalp, as well as plaques and erosions on the vulva and the perianal region were apparent upon admission (23 April 2024). Plaques involving the head, neck, vulva, and perianal region were evident by 10 May 2024. Only pigmentation of the skin at the site of the lesion was observed after 1 month of rituximab therapy (1 July 2024). **(B)** Changes in anti-desmoglein (Dsg) 1 and 3 levels (U/mL) over time. **(C)** Changes in prednisone (mg/day) during the follow-up period. The prednisone dose has now been reduced to 2.5 mg orally per day, and the patient is still under continuous follow-up.

A 36-year-old woman was admitted to our clinic on 23 April 2024, with a history of papulovesicles, pustules, and plaques for 2 months. The lesions initially occurred in her scalp and were diagnosed as contact dermatitis at her first outpatient visit on 21 March 2024, considering the exposure history of hair dye. However, the corresponding treatment was not effective. Subsequently, lesions gradually developed to the vulva and the perianal region. No relevant medication history, previous medical history, and family history was found. Physical examination on admission: papulovesicles, pustules, and erosions were observed on her scalp (**Figure 1A**). Plaques and erosions were visible on both the vulva and the perianal region (**Figure 1A**), with a Pemphigus Disease Area Index (PDAI) score of 23. Over the following 2 weeks, the rash progressed to plaques, involving the head, neck, vulva, and perianal region (**Figure 1A**). There were no other positive findings on systematic physical examination. Laboratory examination revealed the following: anti-desmoglein 1 antibody, 58.72 U/mL (0–20 U/mL); anti-desmoglein 3 antibody, 125.58 U/mL (0–20 U/mL); anti-BP180 antibody, negative; anti-BP230 antibody, negative; syphilis, negative; and HIV, negative. Histopathological findings showed epidermal hyperplasia, acanthosis, formation of intraepidermal eosinophilic microabscesses, intraepidermal acantholysis with acantholytic cells, and infiltration of inflammation cells around the superficial vessels in the dermis (**Figure 1A**). Direct immunofluorescence revealed the intercellular staining of IgG (+), IgA (−), IgM (−), and C3 (−) among epidermal spinous cells (figure not shown). Based on the above findings, we made a diagnosis of pemphigus vegetans.

Following admission, the patient was treated with topical corticosteroids, fusidic acid cream, and potassium permanganate baths for symptomatic treatment. Then, the patient started orally taking prednisone (0.5 mg/kg/day) on 26 April 2024. At the same time, potassium chloride (0.5 g bid), calcium carbonate (1.5 g qd), and pantoprazole (40 mg qd) were administered to prevent the side effects of prednisone. After diagnosis, the prednisone dose was immediately increased to 55 mg/day (1.0 mg/kg/day). After another 2 weeks of treatment, new papulovesicles and erosive lesions formed on the chest and in the armpits, and her PDAI score increased to 31. According to clinical expertise, rituximab was added to the treatment regimen. She was then treated with two doses of rituximab (1,000 mg each) intravenously fortnightly, with the first dose given on 10 May 2024. Her skin lesions showed marked improvement after rituximab therapy (**Figure 1A**). Over a month after first rituximab dose, the PDAI score decreased to 5. Similarly, serum anti-desmoglein 1 and 3 antibody levels decreased markedly after rituximab and remained below 20 during the follow-up period (**Figure 1B**). The frequently reported adverse effects of rituximab, especially infusion reactions, neutropenia, hypoproteinemia, and infections, did not occur in this patient. Unexpectedly, she developed muscle weakness in both arms and legs after over a month of prednisone treatment. A complete workup was carried out. Laboratory examinations, including blood biochemical tests, infection markers, thyroid hormones, and autoantibodies, showed normal results except for a low creatine kinase concentration (22 U/L, normal range: 26–140 U/L). The cranial brain and vertebral magnetic resonance imaging showed no significant abnormality. The needle electromyography showed that motor and sensory conduction velocities of the four limbs were within the normal range. The myopathy in this patient was considered to be secondary to prednisone administration. Given the PVeg was alleviated, the dose of prednisone was immediately tapered (**Figure 1C**), and her muscle weakness gradually attenuated. Fortunately, the patient’s muscle strength recovered when the dose of prednisone was tapered to 12.5 mg/day. The patient’s treatment timeline is shown in **Figure 1**, and she is currently being followed up in the outpatient department.

## Literature review

A search of PubMed was conducted for articles published up to August 2025 using the search terms “pemphigus vegetans AND rituximab”. Reference lists of included studies and review articles were also hand searched for additional articles. Seven articles reporting rituximab treatment for PVeg were finally included (5–11).

## Discussion

PVeg, an autoimmune disease marked by bullae or pustules that erode to form hypertrophic plaques primarily localized to the flexural areas, is a rare variant of PV. Its frequency varies through the literature’s study from 1% to 2% (1). Similar to PV, the primary clinical therapeutic regimen for PVeg is systemic corticosteroid. However, oral corticosteroid alone does not always control the progression of the disease. Several immunosuppressive treatments, such as azathioprine, mycophenolate mofetil, or intravenous immunoglobulins, have been used in combination with glucocorticoids to improve remission rates.

Rituximab is a genetically engineered (chimeric murine/human) monoclonal antibody that targets CD20-positive B cells and induces their depletion *in vivo*, thus reducing the production of autoantibodies. In recent years, numerous case reports and studies have reported on the efficacy of rituximab in treating PV (3, 4). However, because of the rarity of PVeg, there have relatively been a few reports on the use of rituximab for treating PVeg. Here, we reported a rare case of a patient with PVeg who responded poorly to prednisone. The patient was successfully treated by rituximab and followed up for 15 months without relapse.

Furthermore, we went through previous literature and found that only seven prior cases (male-to-female ratio, 3:4; median age, 55 years) of PVeg treated with rituximab have been reported; clinical presentation and management are summarized in **Table 1**. The reported cases were administered prednisone (20 mg/day to 2 mg/kg/day), mycophenolate mofetil, azathioprine, cyclosporine, methotrexate, cyclophosphamide, or doxycycline prior to rituximab. Similar to our patient here, two cases (5, 6) were treated with two 1,000-mg rituximab infusions separated by 2 weeks (the rheumatoid arthritis dose regimen). The remaining five cases received four weekly infusions of rituximab 375 mg/m^2^ (the lymphoma dose regimen). During follow-up, three patients (8, 10, 11) received additional rituximab infusion according to their conditions. Among the seven patients, six (85.7%) responded to rituximab therapy, and there were no fatalities. In one of the cases (5), iatrogenic Kaposi’s sarcoma was observed nearly 3 months after the first dose of rituximab. As we can see, the optimal dosing regimen of rituximab in the management of PVeg remains unknown. Variation exists regarding the efficacy of rituximab treatment, and its long-term efficacy remains unclear. Thus, more clinical studies including a greater number of cases are needed, considering that conducting clinical trials is difficult. To the best of our knowledge, this is the first article to perform literature review on this topic.

**Table 1:** Clinical and immunological features, treatment, and outcome of reported cases of pemphigus vegetans treated with rituximab.

Reference	Patient descriptor	Presentation	Lab findings	Regimens prior to RTX	Dosage	Outcome
Daflaoui et al. (5)	82 M	Multiple purpuric lesions over the legs	Histology: NR DIF: NR	Prednisone 55 mg/day	2 × 1 g (15 days apart)	Iatrogenic Kaposi’s sarcoma induced by RTX and prednisone
Ayoubi et al. (6)	37 M	Paronychia-like changes of the left great toe	Histology: intraepidermal pustules, mild acantholytic clefts, papillary dermal edema, and a mixed dermal infiltrate with eosinophils	MMF (1,000 mg bid)	2 × 1 g (15 days apart)	Resistant to RTX
Ruiz-Villaverde et al. (7)	53 F	Two erythematous plaques on the front and back of both thighs	Histology: acanthosis and papillomatosis in the epidermis with predominantly eosinophilic microabscesses. DIF: deposits of IgG and C3 in the lower part of the epidermis	Prednisone (1 mg/kg/day), Aza, CYC, MTX, MMF	4 × 375 mg/m^2^	The hypertrophic plaques resolved in 45 days. After 1 year of follow-up, the patient remained asymptomatic.
Gregoriou et al. (8)	55 F	NR	Histology: NR DIF: NR	Prednisone 50 mg, CYC	4 × 375 mg/m^2^+375 mg/m^2^ after 6 months	Complete remission off therapy for 12 months.
Barbach et al. (9)	42 F	Vegetative lesions located in the large folds as well as in the hands and feet	Histology: NR DIF: NR	Corticosteroid (2 mg/kg/day), Aza	4 × 375 mg/m^2^	The follow-up without recurrence is 1 year.
Marzano et al. (10)	55 M	Involving scalp, torso, arms, oral, and genital mucosa	Histology: NR DIF: NR	Steroids, Aza, CP	6 × 375 mg/m^2^	A good improvement of the lesions for 2 months.
Iranzo et al. (11)	70 F	Yellowish papulovesicles on the oral mucosa and vegetating pustules on the left inguinal fold and the top of her foot	Histology: focal suprabasal acantholysis and neutrophilic microabscesses with sparse eosinophils in the lower epithelium and extensive lymphocytic infiltrate with isolated eosinophils in the upper and mid-dermis. DIF: weak granular IgA deposition on the keratinocyte cell surfaces in the lower epidermis and stronger IgG deposition on the upper epithelial layers, as well as granular IgA, IgG,and C3 deposition along the BMZ.	Prednisone 20 mg/day, Aza, doxycycline	4 × 375 mg/m^2^+4 × 375 mg/m^2^ after 7 months	After 2 months, cutaneous lesions disappeared and oral mucosal lesions improved.

Aza, azathioprine; CP, cyclophosphamide; CYC, cyclosporine; DIF, direct immunofluorescence; F, female; IgG, immunoglobulin G; M, male; MMF, mycophenolate mofetil; MTX, methotrexate; NR, not reported; RTX, rituximab.

Steroid myopathy is one of the well-established side effects of glucocorticoids. Long-term application of glucocorticoids in high dose is required for some patients with PV, which inevitably leads to an increased risk of steroid myopathy (12). However, accurate measurement of steroid myopathy is difficult because of the lack of specific and sensitive diagnostic tests, and muscle strength is rarely evaluated in dermatological practice. Thus, there are limited data on steroid myopathy in patients with pemphigus. In this paper, we reported a patient with PVeg who developed muscle weakness after approximately 1 month of treatment with prednisone (1.0 mg/kg/day). Decreased myodynamia was found in the upper and lower limbs (Grade 4, MRC scale). However, blood tests, imaging examinations, and electromyography revealed no significant abnormalities. Thus, immune-mediated myopathies (e.g., polymyositis and dermatomyositis), infectious myopathies, neoplastic myopathies, myasthenia gravis, and endocrine myopathy (e.g., thyroid disorders) were ruled out. Based on this analysis, the drug-induced myopathy was suspected. Fortunately, the dose of prednisone was rapidly tapered to 12.5 mg every day and the patient’s muscle strength recovered, which provides support for the diagnosis of steroid myopathy. Unfortunately, the patient refused muscle biopsy, which precluded this study from diagnosing steroid myopathy more objectively. The potential role of rituximab in causing muscle weakness in this patient remains unclear. The rituximab-related myopathy is rarely reported, and it is important to note that several observational studies have demonstrated rituximab’s effectiveness in treating myositis and related myopathies (13, 14). In this case report, rituximab therapy not only helped improve the patient’s clinical condition but also allowed for the rapid tapering of prednisone doses, reducing the overall corticosteroid burden and minimizing associated side effects.

## Conclusion

In summary, we present a rare case of PVeg successfully treated by rituximab and provide the first brief review of rituximab in the management of PVeg. In addition, this case report aims to increase awareness regarding the complication of steroid myopathy among dermatologists.

## Data Availability

The original contributions presented in the study are included in the article/Supplementary Material. Further inquiries can be directed to the corresponding author.

## References

[1] Zaraa I Sellami A Bouguerra C Sellami MK Chelly I Zitouna M . Pemphigus vegetans: a clinical, histological, immunopathological and prognostic study. J Eur Acad Dermatol Venereol: JEADV. (2011) 25:1160–7. doi: 10.1111/j.1468-3083.2010.03939.x, PMID: 21198951

[2] Ruocco V Ruocco E Caccavale S Gambardella A Lo Schiavo A . Pemphigus vegetans of the folds (intertriginous areas). Clinics Dermatol. (2015) 33:471–6. doi: 10.1016/j.clindermatol.2015.04.011, PMID: 26051064

[3] Joly P Maho-Vaillant M Prost-Squarcioni C Hebert V Houivet E Calbo S . First-line rituximab combined with short-term prednisone versus prednisone alone for the treatment of pemphigus (Ritux 3): a prospective, multicentre, parallel-group, open-label randomised trial. Lancet (London England). (2017) 389:2031–40. doi: 10.1016/S0140-6736(17)30070-3, PMID: 28342637

[4] Cao S Yang B Wang Z Liu T Sun Y Zhang Z . Efficacy, safety, and B-cell depletion capacity of 3 rituximab dosing regimens in the treatment of moderate-to-severe pemphigus vulgaris and pemphigus foliaceus: A 52-week clinical trial. J Am Acad Dermatol. (2025) 93:634–43. doi: 10.1016/j.jaad.2025.05.1374, PMID: 40374120

[5] Daflaoui H Saddouk H Ouadi I Zizi N Dikhaye S . Iatrogenic Kaposi’s sarcoma induced by rituximab and corticosteroid treatment for pemphigus vegetans in an HIV-negative patient. Indian J Dermatol Venereol Leprol. (2022) 88:409–12. doi: 10.25259/IJDVL_688_2021, PMID: 35434989

[6] Ayoubi N Rudnick E Motaparthi K . Pemphigus vegetans with paronychia-like changes resistant to rituximab therapy. Dermatol Ther. (2020) 33:e13515. doi: 10.1111/dth.13515, PMID: 32367637

[7] Ruiz-Villaverde R Galán-Gutierrez M Sanchez-Cano D . Treatment of pemphigus vegetans to rituximab refractory to conventional therapy. Acta Dermatovenerol Croatica: ADC. (2014) 22:221–3., PMID: 25230067

[8] Gregoriou S Giatrakou S Theodoropoulos K Katoulis A Loumou P Toumbis-Ioannou E . Pilot study of 19 patients with severe pemphigus: prophylactic treatment with rituximab does not appear to be beneficial. Dermatol (Basel Switzerland). (2014) 228:158–65. doi: 10.1159/000357031, PMID: 24557145

[9] Barbach Y Baybay H Mrabat S Chaouche M Elloudi S Mernissi FZ . Unexpected positive outcome following rituximab treatment in a patient with pemphigus vegetans resistant to conventional therapies: a case report. Pan Afr Med J. (2019) 32:101. doi: 10.11604/pamj.2019.32.101.16743, PMID: 31223391 PMC6560954

[10] Marzano AV Fanoni D Venegoni L Berti E Caputo R . Treatment of refractory pemphigus with the anti-CD20 monoclonal antibody (rituximab). Dermatol (Basel Switzerland). (2007) 214:310–8. doi: 10.1159/000099591, PMID: 17460402

[11] Iranzo P Ishii N Hashimoto T Alsina-Gibert M . Nonclassical pemphigus with exclusively IgG anti-desmocollin 3-specific antibodies. Australas J Dermatol. (2019) 60:e217–e9. doi: 10.1111/ajd.12991, PMID: 30671942

[12] He A Koszegi B Uzun S Bilgic A Bozca BC Yang B . Autoimmune blistering diseases treated with glucocorticoids: An international study of steroid-induced myopathy. J Eur Acad Dermatol Venereol: JEADV. (2025) 39:340–9. doi: 10.1111/jdv.20149, PMID: 38818849 PMC11761005

[13] Gilaberte S Rua J Isenberg D . Adverse events of treatment with rituximab in patients with myositis. Rheumatol (Oxford England). (2023) 62:e16–e7. doi: 10.1093/rheumatology/keac398, PMID: 35809059

[14] Zhen C Hou Y Zhao B Ma X Dai T Yan C . Efficacy and safety of rituximab treatment in patients with idiopathic inflammatory myopathies: A systematic review and meta-analysis. Front Immunol. (2022) 13:1051609. doi: 10.3389/fimmu.2022.1051609, PMID: 36578492 PMC9791086

